# New Antiproliferative Triflavanone from *Thymelaea hirsuta*—Isolation, Structure Elucidation and Molecular Docking Studies

**DOI:** 10.3390/molecules26030739

**Published:** 2021-01-31

**Authors:** Sameh S. Elhady, Reda F. A. Abdelhameed, Mayada M. El-Ayouty, Amany K. Ibrahim, Eman S. Habib, Mohamed S. Elgawish, Hashim A. Hassanean, Martin K. Safo, Mohamed S. Nafie, Safwat A. Ahmed

**Affiliations:** 1 Department of Natural Products, Faculty of Pharmacy, King Abdulaziz University, Jeddah 21589, Saudi Arabia; ssahmed@kau.edu.sa; 2 Department of Pharmacognosy, Faculty of Pharmacy, Suez Canal University, Ismailia 41522, Egypt; omarreda_70@yahoo.com (R.F.A.A.); maya.badawy159@gmail.com (M.M.E.-A.); am_kamal66@yahoo.com (A.K.I.); emy_197@hotmail.com (E.S.H.); hashem_omar@pharm.suez.edu.eg (H.A.H.); 3 Department of Pharmacognosy, Faculty of Pharmacy, Sinai University, El-Arish 45511, Egypt; 4 Department of Medicinal Chemistry, Faculty of Pharmacy, Suez Canal University, Ismailia 41522, Egypt; mohamed_elgawish@pharm.suez.edu.eg; 5 Department of Medicinal Chemistry, School of Pharmacy and Institute for Structural Biology, Drug Discovery and Development, Virginia Commonwealth University, Richmond, VA 23219, USA; msafo@vcu.edu; 6 Department of Chemistry, Faculty of Science, Suez Canal University, Ismailia 41522, Egypt; mohamed_nafie@science.suez.edu.eg

**Keywords:** *Thymelaea hirsuta*, flavanone, dicoumarinyl ether, cytotoxicity, molecular docking

## Abstract

In this study isolates from *Thymelaea hirsuta*, a wild plant from the Sinai Peninsula of Egypt, were identified and their selective cytotoxicity levels were evaluated. Phytochemical examination of the ethyl acetate (EtOAc) fraction of the methanolic (MeOH) extract of the plant led to the isolation of a new triflavanone compound (**1**), in addition to the isolation of nine previously reported compounds. These included five dicoumarinyl ethers found in *Thymelaea*: daphnoretin methyl ether (**2**), rutamontine (**3**), neodaphnoretin (**4**), acetyldaphnoretin (**5**), and edgeworthin (**6**); two flavonoids: genkwanin (**7**) and *trans*-tiliroside (**8**); *p*-hydroxy benzoic acid (**9**) and *β* sitosterol glucoside (**10**). Eight of the isolated compounds were tested for in vitro cytotoxicity against Vero and HepG2 cell lines using a sulforhodamine-B (SRB) assay. Compounds **1**, **2** and **5** exhibited remarkable cytotoxic activities against HepG2 cells, with IC_50_ values of 8.6, 12.3 and 9.4 μM, respectively, yet these compounds exhibited non-toxic activities against the Vero cells. Additionally, compound **1** further exhibited promising cytotoxic activity against both MCF-7 and HCT-116 cells, with IC_50_ values of 4.26 and 9.6 μM, respectively. Compound **1** significantly stimulated apoptotic breast cancer cell death, resulting in a 14.97-fold increase and arresting 40.57% of the cell population at the Pre-G1 stage of the cell cycle. Finally, its apoptosis-inducing activity was further validated through activation of BAX and caspase-9, and inhibition of BCL2 levels. In silico molecular docking experiments revealed a good binding mode profile of the isolates towards Ras activation/pathway mitogen-activated protein kinase (Ras/MAPK); a common molecular pathway in the development and progression of liver tumors.

## 1. Introduction

Cancer is a leading cause of morbidity and mortality. Years of research attempting to find new and effective anticancer agents has not only improved survival and the quality of life of patients, but has also significantly reduced the need for surgical procedures [1]. Natural products derived from plants have served as an important source of pharmacologic agents for several diseases [2], including cancer [3]. An example is the Thymelaeaceae family of plants, that have been shown to exhibit many pharmacological activities [4,5,6,7,8,9,10,11,12,13,14,15,16,17,18]. For example, extracts of *Daphne giraldii* [4] and *Wikstroemia indica* [5] from the Thymelaeaceae family of plants are known to contain various pharmacologically active constituents, including: flavonoids [6], coumarins [7], diterpenoids [8], lignans, volatile oils and polysaccharides [9] that have been used for the tretament of several ailments. The plants *Lasiosiphon eriocephalus* [10], *Daphne mezereum* [11], and *Daphne acutiloba* [12] have also been shown to have cytotoxic activities against different types of cancer. Another important member of the Thymelaeaceae family is *T. hirsute*, a native plant of North Africa. *T. hirsute* contains flavonoids, coumarins, tannins, and saponins [13], and has been demonstarted to have antiviral activity against HIV-1 [14], antioxidant activity [15], antidiabetic and antihypertensive activities [16]. Other studies have also shown isolates from *T. hirsuta* to have significant cytotoxicity against hepatocellular carcinoma (HCC) [17], which is one of the leading causes of cancer deaths worldwide, with a rapidly increasing incidence rate in Egypt [18].

In a continuing effort to discover bioactive metabolites from Egyptian plants [17,19], our group focused on the isolation of active constituents from the EtOAc fraction of the total MeOH extract from the whole *T. hirsute* plant. Different spectroscopic techniques were then used to identify the isolated compounds. The cytotoxic activities of eight of the isolated compounds were tested against cancer and normal cell lines using a sulforhodamine-B (SRB) assay, following the method reported by Vichai and Kirtikara [20]. Molecular docking experiments were also conducted to gain insight into the molecular mechanism of the isolates.

## 2. Results and Discussion

### 2.1. Isolation of Compounds ***1***–***10***

**Figure 1:**
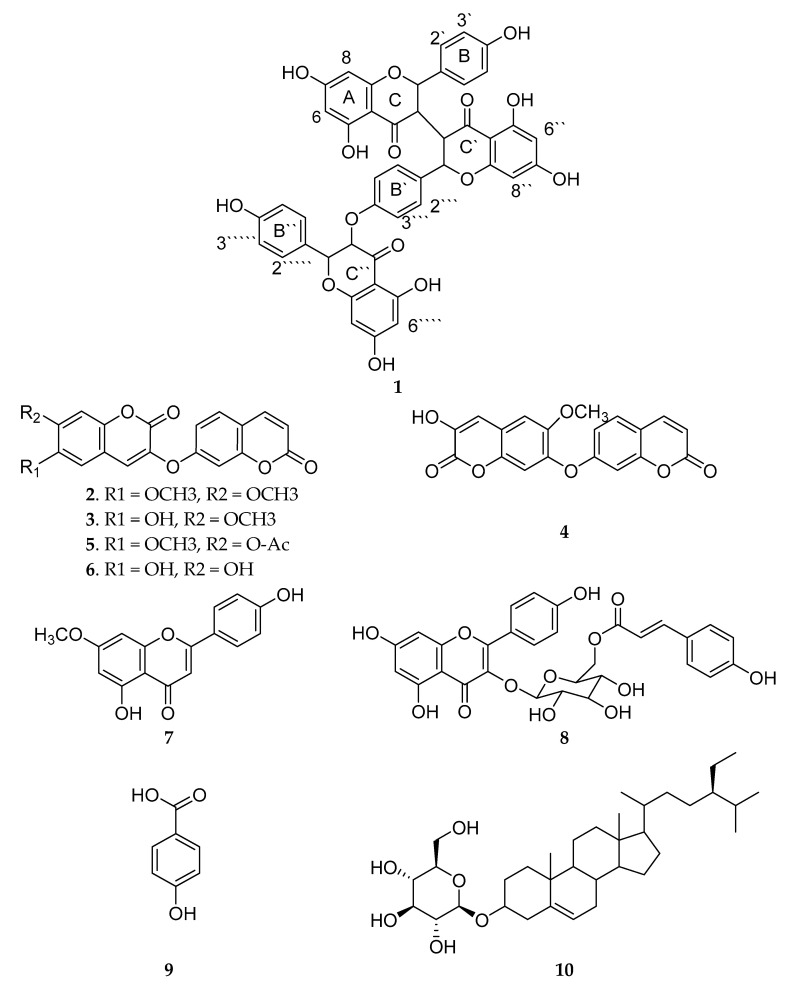
Structure of isolated compounds **1**–**10**.

Using different chromatographic isolation techniques, ten compounds **1**–**10** (Figure 1) were isolated and purified from the EtOAc fraction of the MeOH extract of *T. hirsuta*. One of the isolated compounds (**1**) was identified as new; five were dicoumarinyl ethers, including daphnoretin methyl ether (**2**), rutamontine (**3**), neodaphnoretin (**4**), acetyldaphnoretin (**5**), and edgeworthin (**6**); two were flavonoids, including genkwanin (**7**) and *trans*-tiliroside (**8**); while the remaining two were *p*-hydroxy benzoic acid (**9**) and β sitosterol glucoside (**10**). Spectroscopic methods, including LC-MS/MS, 1D and 2D NMR were used for the characterization and interpretation of the isolated compounds, and to allow comparisons with published data.

### 2.2. Identification of Compounds ***1***–***10***

Compound **1** was isolated as a yellow powder and its molecular formula was determined as C_45_H_32_O_15_ by using an LC-MS/MS (Figure S1) in negative mode, which showed a molecular ion peak at *m/z* 811.1644 [M − H]^−^, and calculated as 811.1663 [M − H]^−^. The mass spectrum (Figure S1) also showed a fragment ion *m/z* of 541.1134, which represented a neochamaejasmin B fragment ion, in addition to a fragment ion *m/z* of 415.0932 that represented the loss of a phloroglucinol unit (C_6_H_6_O_3_) from the neochamaejasmin B fragment ion [21], indicating that neochamaejasmin B is part of compound **1**.

The ^1^H-NMR spectrum of **1** (Table 1, Figures S2–S4) displayed signals of three protons of H-2 (*δ*_H_ 5.47, 1H, *d*, *J* = 4.5 Hz), H-2`` (*δ_H_* 5.14, 1H, *d*, *J* = 9.0 Hz), H-2```` (*δ_H_* 4.96, 1H, *m*), and three protons of H-3 (*δ_H_* 3.15, 1H, *m*), H-3`` (*δ_H_* 3.32, 1H, *m*) H-3```` (*δ_H_* 5.89, 1H, *d*, *J* = 2.1 Hz). The aromatic proton signals (*δ_H_* 5.75–7.11, 18H) indicated the presence of three sets of typical 5,7-dioxygenated A rings (*δ_H_* 5.76, 5.79, each 1H, *d*, *J* = 2.1 Hz; *δ_H_* 5.87 (1H, *d*, *J* = 2.1), 5.98 (1H, *m*); *δ_H_* 5.75, 2H, *s*) and three sets of para-oxygenated B rings (*δ_H_* 7.11, 6.64, each 2H, *d*, *J* = 8.7 Hz and 8.4 Hz, respectively; *δ_H_* 6.92, 6.79, each 2H, *d*, *J* = 8.4 Hz; *δ_H_* 7.01, 6.78, each 2H, *d*, *J* = 8.4 Hz and 8.7 Hz, respectively).

The ^13^C-NMR data (Table 1) showed the presence of three carbonyls (*δ_C_* 198.9, 196.6, 197.3). The structure of compound **1** was therefore determined to consist of three flavanone units. By comparing the spectroscopic data: ^1^H-NMR, ^13^C-NMR and HMBC spectra (Figures S2–S7), with the published data of mithnin [17], neochamaejasmin B [22], and 3,3``-biflavanones [23,24,25], compound **1** was found to be a triflavanone. By comparing the *J* values (H-2, *J* = 4.5 Hz and H-2`` *J* = 9.0 Hz) with those of mithnin [17], and 3,3``-biflavanones [23,24,25], C-2/C-3 and C-2``/C-3`` of the first and second flavanone regions of **1**, were found to have a cis-trans geometry.

**Figure 2:**
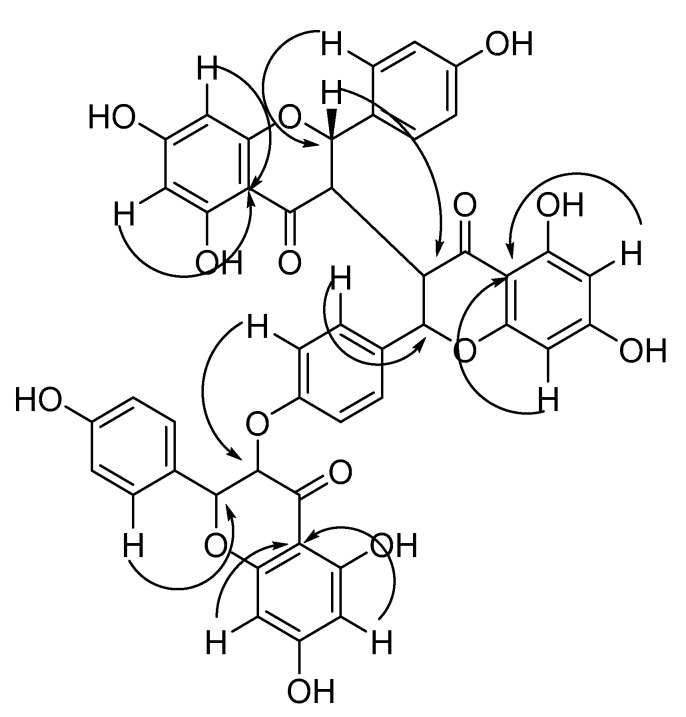
HMBC correlations of compound **1**.

The first flavanone is linked to the second flavanone at the C-3 and C-3`` positions, which was confirmed by comparison of the ^1^H- and ^13^C-NMR data of **1** with known 3,3``-biflavanones [26] and mithnin [17]. Further confirmation came from comparison with the HMBC spectrum (Figures S6 and S7), which showed a correlation between H-2 (δ_H_ 5.54) and C-3`` (*δ*_C_ 51.2). The second flavanone is linked to the third flavanone by the C3```` of the third flavanone and the hydroxyl group in the para position of ring B` of the second flavanone. This correlation was confirmed using HMBC, as the C3```` at *δ*_C_ 97.8 correlated with the H-3```, H-5``` at *δ*_H_ 6.79. The linkage between the B ring and the C ring for each of the three flavanones was identified in the HMBC, and for each, the B ring was located at C2 of the C ring. This was determined as the HMBC spectrum showed that H-2` and H-6`(*δ*_H_ 7.11) correlated with C-2 (δ_C_ 81.8), H-2``` and H-6``` (*δ*_H_ 6.92) correlated with C-2`` (*δ*_C_ 83.5), and H-2````` and H-6````` (*δ*_H_ 7.01) correlated with C-2```` (*δ*_C_ 82.8) (Figure 2). Based on the above analysis, compound **1** was identified as a new compound: 2-(4-((5,7-dihydroxy-2-(4-hydroxyphenyl)-4-oxochroman-3-yl)oxy)phenyl)-5,5′,7,7′-tetrahydroxy-2′-(4 hydroxyphenyl)-[3,3′-bichromane]-4,4′-dione.

Compounds **2**–**10** (Figure 1) were identified using different spectroscopic techniques and by comparing the resulting data with the data published in the literature. The compounds were identified as daphnoretin methyl ether (**2**) [27], rutamontine (**3**) [28], neodaphnoretin (**4**) [29], acetyl daphnoretin (**5**) [30], edgeworthin (**6**) [27], genkwanin (**7**) [31,32], *trans*-tiliroside (**8**) [33], *p*-Hydroxybenzoic acid (**9**) [34] and *β*-sitosterol glucoside (**10**) [35].

### 2.3. Cytotoxic Activities

**Figure 3:**
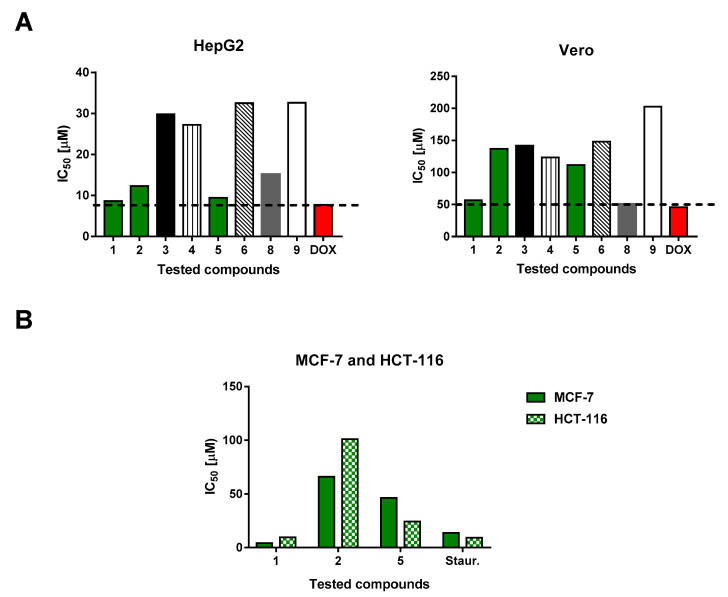
(**A**) Screening of the IC_50_ values of the tested compounds against the HepG2 cells and Vero normal cells, and (**B**) IC_50_ values of the most active compounds **1**, **2**, **5** and staurosporine (control) against both MCF-7 and HCT-116 cell lines. IC_50_ values were calculated using the non-linear regression curve fit of the percentages of cell viabilities with the tested concentrations.

Two of the isolates, Genkwanin (**7**) and *β* sitosterol glucoside (**10**), had already been tested for their cytotoxic activity against HepG2 cancer cell line [31,36], therefore, we only screened for the cytotoxicity of the other eight isolated compounds **1**–**6**, **8** and **9** against liver HepG2 and normal Vero cells (Table 2, Figure 3).

As shown in Figure 3, compounds **1**, **2** and **5** exhibited remarkable cytotoxic activities against HepG2 cells with IC_50_ values of 8.6, 12.3 and 9.4 μM, respectively. However, they exhibited non-toxic activities against the Vero cells (IC_50_ > 50 μM). Following, the three compounds were further tested against the cancer cell lines, MCF-7 and HCT-116. Compounds **1**, **2** and **5** showed cytotoxic activities against MCF-7 cells, with IC_50_ values of 4.26, 66.14 and 46.38 μM, respectively, and against HCT-116 cells, with IC_50_ values of 9.60, 100.90 and 24.35 μM, respectively. Compound **1** clearly showed very promising cytotoxic activity against all five tested cancer cell lines, and was consequently chosen for further investigation to determine its apoptosis induction ability in MCF-7 cells.

### 2.4. Cell Cycle Analysis

#### 2.4.1. Annexin V/PI Staining

**Figure 4:**
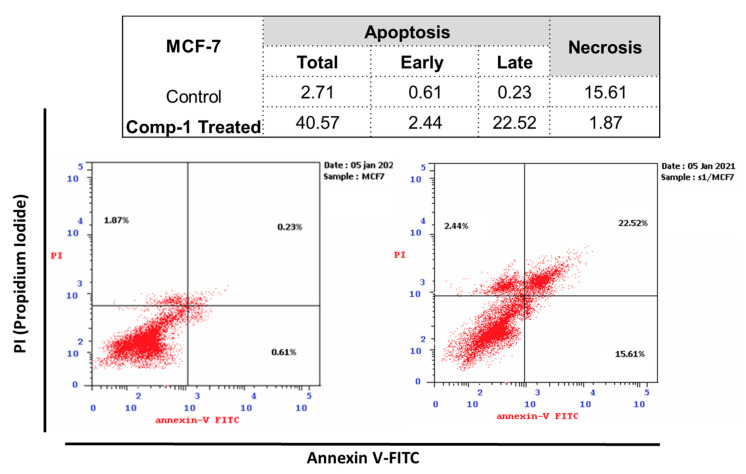
Cryptographs of annexin V/Propidium Iodide staining of untreated and compound **1**-treated MCF-7 cells (IC_50_ = 4.26 μM, 48 h), Q2-1 (necrosis, AV-/PI+), Q2-2 (late apoptotic cells, AV+/PI+), Q2-3 (normal cells, AV-/PI-), Q2-4 (early apoptotic cells, AV+/PI-), with summarized table for the percentage of cell population in early, late apoptotic, and necrotic cell death.

The promising anti-cytotoxic activity of Compound 1 prompted us to further investigate this compound for its apoptosis-inducing activity in MCF-7 cells. After treatment, cells were subjected to flow cytometric analysis of annexin V/PI staining with cell cycle analysis to determine the cell population in different cell cycle phases. As seen in Figure 4, compound **1** significantly stimulated apoptotic breast cancer cell death, demonstrating a 14.97-fold cell death increase. It also increased the apoptosis ratio by 40.57%, compared to 2.71% for the control. It increased the induction of early apoptosis by 2.44% compared to 0.61% for the control, and late apoptosis by 22.52% compared to 0.23% for the control. Our results are in accordance with our previous studies [37,38] of apoptosis induction with various isolates from medicinal plants.

#### 2.4.2. Cell Cycle Analysis

**Figure 5:**
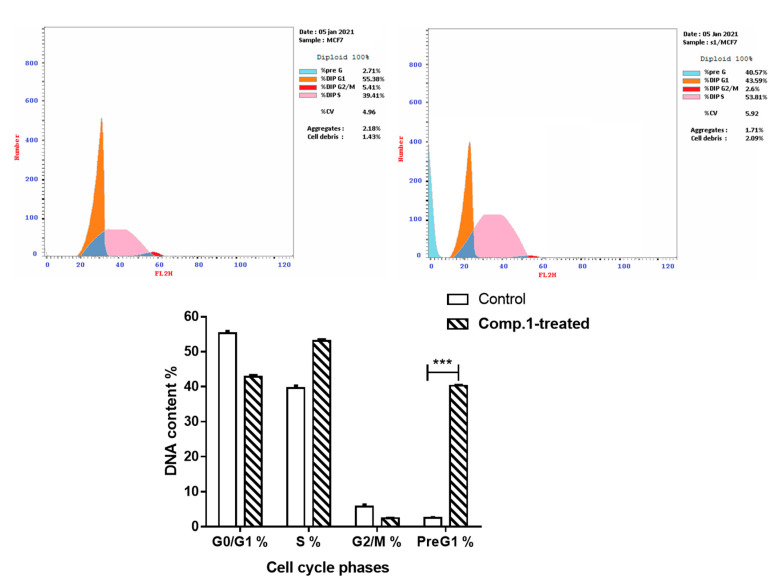
Histogram DNA content flow cytometry aided cell cycle analysis of untreated and compound **1**-treated MCF-7 cells (IC_50_ = 4.26 μM, 48 h), with Bar-representation of the percentage of cell population at each stage of cell cycle G0/G1, S, G2/M, and Pre G1. *** *p* ≤ 0.001 are significant different

Cell cycle analysis is an important test that determines the percentages of the cell population that are in each cell phase.. In this studyMCF-7 cancer cells were treated with compound **1**, and then subjected to DNA flow cytometry to determine the stage where cell proliferation was arrested in the cell cycle. As seen in Figure 5, compound treatment significantly increased the Pre-G1 population by 40.57%, compared to 2.71% for the control. In contrast, there was no significant change in the cell population in the G0/G1, S or G2/M cell phase.

### 2.5. Enzymatic Assays for Apoptotic Markers

**Figure 6:**
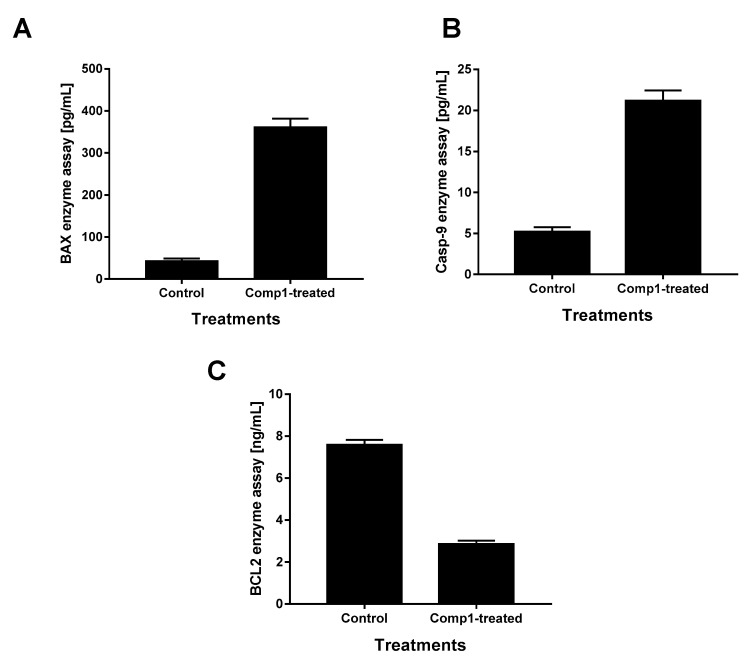
Enzymatic ELISA assays for apoptotic markers; (**A**) BAX, (**B**) caspase-9, and (**C**) BCL2 enzymes for both untreated and compound **1**-treated MCF-7 cells (IC_50_ = 4.26 μM, 48 h). Data illustrate the average of three independent experimental runs.

For further investigation of compound 1 apoptotic behavior, treated and untreated MCF-7 cells with compound **1** were analyzed for the activities of pro-apoptotic BAX and caspase-9, and the anti-apoptotic BCL2. As shown in Figure 6, treatment with compound **1** significantly increased BAX level (362 pg/mL) compared to the control (43.6 pg/mL), as well as caspase-9 level (21.14 pg/mL) compared to the control (5.16 pg/mL), while it decreased BCL2 level to 2.85 ng/mL compared to the control (7.59 ng/mL). Hence, the enzymatic activity of both BAX and caspase-9 and the inhibition of BCL2 represent significant evidence of the induction of apoptosis in the MCF-7 cells. 

### 2.6. Molecular Docking 

Molecular docking simulation is an important approach for predicting the coupling of a substrate with its receptor. The Ras activation/pathway mitogen-activated protein kinase (Ras/MAPK) pathway has been identified as being capable of inducing tumor initiation, development, and progression in HCC and thus, it represents a promising target for cancer drug development. As noted above, several substituted coumarins have been shown to have potent anti-hepatocellular carcinoma activities through their targeting of the Ras/MAPK pathway [39,40,41]. With the exception of two of the compounds (Compounds **9** and **10**), the rest of the identified compounds are coumarins, which prompted us to evaluate the binding modes of the compounds with MAPK.

Docking experiments were used to map the binding pose of isolates with MAPK and to predict their binding affinities. The docking scores and binding free energies of the lowest energy pose of the compounds in the MAPK active site—using the Schrodinger Glide module—are shown in Table 3. MAPK is an ATP-dependent protein kinase, in which the hydrophobic pocket (the ATP-binding pocket) is rich in alanine, valine, isoleucine, and leucine amino acids.

**Figure 7:**
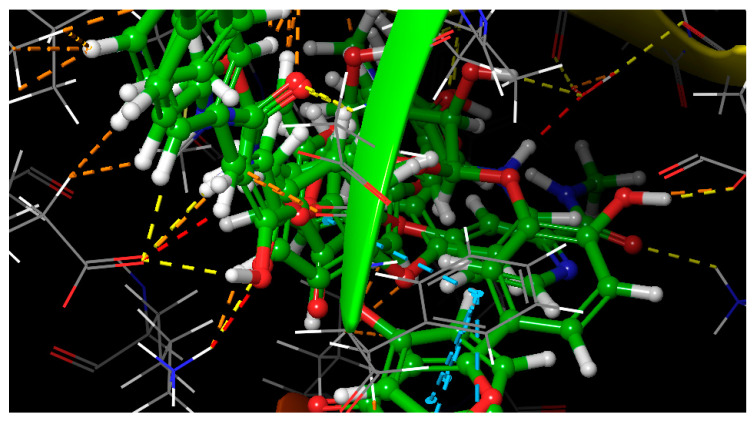
3D binding mode and overlay of isolates, rutin and Sorafenib in mitogen-activated protein kinase (MAPK) active domain (PDB 5EKN). Image shows hydrogen bonding as a yellow dotted line, pi-pi staking interactions as a cyan dotted line, and hydrophobic interactions as an orange dotted line.

The docking study suggested that the isolates interact with several main amino acid residues at the MAPK active site through hydrophobic contacts (orange lines), H-bonding (yellow dotted lines), and electrostatic interactions (cyan color) (Figure 7).

The MAPK protein is capable of forming hydrogen-bond and hydrophobic interactions with ligands at the active site as shown in Figure S8. Our modeling suggests that nearly all of the isolates could form hydrophobic and π-π stack interactions with PHE169. The hydroxyl and ketonic groups of the isolates also should contribute to hydrogen-bond interactions with several amino acids, including TYR36, LYS54, LYS116, and ASP113. These interactions are expected to allow for tight binding of the isolates to MAPK (Figures S9–S11). Hydroxylated-chromones mimic the adenine ring of ATP and occupied the hydrophobic pocket in the hinge region, forming hydrogen-bond interactions with MET 110, and LYS116. 

To choose the best docking pose with the lowest energy, the docking procedure was repeated several times. The results of the docking protocols were similar, suggesting the glide docking technique was highly reproducible. The isolates’ extra precise glide docking with MAPK showed a good docking score of −8.55 and −8.34 kJ/mol with a glide Emodel value of −58.78 and −100.70 kcal mol^−1^, especially for compounds **7** and **8**, respectively. The reliability of the planned docking protocol was evaluated by assessing the interactions of the most widely recognized flavonoid of modulation activity, rutin, with MAPK [42]. The isolates showed a higher binding affinity and binding energy compared to rutin, which showed the same pose (Figure S12) as the isolates, achieving encouraging docking ratings of −7.32 kJ/mol and a glide Emodel value of −69.18 kcal mol^−1^. Furthermore, the isolates were compared to sorafenib, an oral Ras/MAPK inhibitor that is used as a therapy for advanced liver carcinoma [43]. Sorafenib occupies the active site of MAPK (Figure S13) in the same pose as the isolates and shows hydrogen-bond interactions with GLU72, LYS116, and ASP169, achieving a comparable docking score (−8.37 kJ/mol) and binding energy (−81.1 kcal mol^−1^) to those of our isolates.

## 3. Materials and Methods 

### 3.1. Plant Material

The whole plant of *T. hirsuta* was collected from the Sinai Peninsula of Egypt and was authenticated by the Botany department, faculty of Science, Suez Canal University, Egypt. The voucher specimen (No. SAA-162) was stored at the Pharmacognosy Herbarium, Suez Canal University, Ismailia, Egypt.

### 3.2. Extraction and Isolation 

The plant (10 kg) was air dried, grounded, and the resulting powder was macerated using methanol at room temperature to yield 250 g of dry extract. Fractionation using vacuum liquid chromatography (VLC) with gradient elution led to the separation of different fractions. Using a hexane, ethyl acetate, and methanol gradient elution, the EtOAc fraction was subjected to column chromatography of silica gel type. Five fractions were obtained and subjected to different chromatographic separation techniques, including silica gel and sephadex LH-20 column chromatography.

For the fraction of 10% methanol in ethyl acetate, 9 g was purified using sephadex LH-20 with a mixture of methanol and chloroform (1:1) as the isocratic mobile phase to yield **1** (23 mg). For the fraction of 60% ethyl acetate in hexane, 7 g was purified using silica gel column chromatography and isocratic elution to yield **2** (17 mg), **3** (25 mg), and **4** (23 mg). For the fraction of 70% ethyl acetate in hexane, 5 g was purified using silica gel column chromatography and isocratic elution to yield **5** (27 mg) and **6** (14 mg). For the fraction of 40% ethyl acetate in hexane, 5 g was purified using sephadex LH-20 with a mixture of methanol and chloroform (1:1) as the isocratic mobile phase to yield **7** (15 mg) and **9** (10 mg). For the fraction of 100% ethyl acetate, 8 g was purified using sephadex LH-20 with mixture of methanol and chloroform (1:1) as the isocratic mobile phase to yield **8** (30 mg) and **10** (35 mg).

### 3.3. Cytotoxic Activity

#### 3.3.1. Cell Lines

The cell lines used in this study were purchased from the National Cancer Institute, Cairo, Egypt and maintained in Dulbecco’s Modified Eagle Medium/F-12 (DMEM⁄F12, Sigma-Aldrich, USA), supplemented with 2 mM l-glutamine (Lonza, Belgium) and 10% fetal bovine serum (FBS, Sigma-Aldrich, MO, USA), 1% penicillin-streptomycin (Lonza, Belgium). All cells were cultured following routine tissue culture work, and treated with serial concentrations of the compounds for 48 h. Absorbance was subsequently measured (at 570 nm) using ELISA microplate reader (BIO-RAD, model iMark, Japan). The viability was calculated relative to a control and the IC_50_ values were determined using the non-linear regression curve fit, as previously reported in [44]

#### 3.3.2. Procedure

The cytotoxic activities of the isolated compounds against HepG2, VERO E6, MCF-7 and HCT-116 cells were tested using the sulforhodamine-B (SRB) assay [20] and the results are represented in figure e and Table 2.

#### 3.3.3. Investigation of Apoptosis 

##### Annexin V/PI Staining and Cell Cycle Analysis 

Apoptosis rate in cells was quantified using annexin V-FITC (BD Pharmingen, San Diego, CA, USA). MCF-7 cells were seeded into 6-well culture plates (3–5 × 10^5^ cells/well) and incubated overnight. The cells were then treated with compound **1** for 48 h. Next, media supernatants and cells were collected and rinsed with ice-cold PBS. The next step was suspending the cells in 100 µL of annexin binding buffer solution 25 mM CaCl_2_, 1.4 M NaCl, and 0.1 M Hepes/NaOH, pH 7.4 and incubation with annexin V-FITC solution (1:100) and propidium iodide (PI) at a concentration of 10 µg/mL in the dark for 30 min. Stained cells were then acquired by a Cytoflex FACS machine. Data were analyzed using cytExpert software v.2.3. [45,46,47]

##### Enzymatic Assays for Apoptotic Markers

To further investigate the apoptotic pathway of compound **1** in both untreated and treated MCF-7 cells, we investigated the enzymatic assays for apoptotic markers BAX (EIA-4487, DRG International^®^, Springfield, NJ, USA) and caspase-9 (EIA-4860, DRG International^®^, USA) as proapoptotic genes and BCL2 (Cat. No. 99-0042, Zymed^®^, CA, USA) as the anti-apoptotic gene, following the manufacturer’s instructions.

### 3.4. Molecular Modeling

A molecular modeling analysis was conducted using the Glide docking method implemented in the molecular modeling software Schrodinger-10.1. The X-ray crystal structure of the MAPK enzyme’s catalytic domain inhibitor complex (PDB ID: 5EKN; 2.6 Å) was obtained from the Protein Data Bank (PDB) (http://www.rcsb.org/pdb). Using the protein preparation wizard and the OPLS-2005 force field, the MAPK-ligand complex was optimized for the Glide docking calculations. Crystallographic water was extracted, if present, and hydrogen was added to the structure corresponding to pH 7.0; the most likely positions of hydroxyl and thiol hydrogen atoms, taking into account the correct ionization conditions for both the basic and acidic amino acid residues of the protein. The protein charge and protonation state were then modified by the protein assignment script, and the protein-inhibitor complex was subjected to energy minimization until the non-hydrogen atom average root mean square deviation (RMSD) exceeded 0.3 Å to trigger steric clashes using the OPLS-2005 force field. 

The 3D structure of the N58 was constructed using the ligand preparation wizard and optimized with the Maestro build stand. For each input molecule the ligand preparation feature produces many low-energy 3D structures with different ionization states, tautomers, stereo-chemistries, and ring conformations. Partial atomic charges were attributed to N58 using the force-field of OPLS-2005, and potential ionization states were created at a pH of 7. The van der Waal radii of the receptor atoms were multiplied by 0.8 with a partial atomic charge of 0.15 to minimize the potential for non-polar parts of the receiver. At the center of the active site, a grid box with coordinates X = 10, Y = 10 and Z = 10 was generated. The resulting ligand structures were further optimized by energy minimization until the RMSD limit of 0.01 Å was reached. Having ensured that the MAPK enzyme and inhibitor molecules were in the appropriate form, the properties and shape and the active site of MAPK were characterized using the “grid generation row” in Glide. In the final step, using the optimized protein-ligand geometries, the separated compounds were docked inside the active site of MAPK. The extra precision (XP) glide scoring function, which flexibly docks ligands, was applied to rank the docking poses and to evaluate the binding affinities between the protein–ligand. Maestro’s Pose Viewer versatility was used to envision and evaluate the key elements of the ligand–receptor interaction. Using a glide score function, the final best-docked structure with the lowest energy was selected for further experiments. The inhibitor was removed from the MAPK enzyme crystal structure and then re-docked using the above step to evaluate the accuracy and precision of the docking protocol established [48,49].

## 4. Conclusions

The phytochemical examination of the EtOAc fraction of the MeOH extract of *T. hirsuta* in the present study led to the isolation of one new compound (**1**) named 2-(4-((5,7-dihydroxy-2-(4-hydroxyphenyl)-4-oxochroman-3-yl)oxy)phenyl)-5,5′,7,7′-tetrahydroxy-2′-(4 hydroxyphenyl)-[3,3′-bichromane]-4,4′-dione; as well as nine other compounds, including five first reported dicoumarinyl ether compounds which were identified as daphnoretin methyl ether (**2**), rutamontine (**3**), neodaphnoretin (**4**), acetyldaphnoretin (**5**), and edgeworthin (**6**), and four previously isolated compounds, which were identified as genkwanin (**7**), *trans*-tiliroside (**8**), *p*-hydroxy benzoic acid (**9**) and *β* sitosterol glucoside (**10**). Compounds **1**, **2** and **5** exhibited remarkable cytotoxic activities against HepG2 cells, with IC_50_ values of 8.6, 12.3 and 9.4 μM, respectively. However, they exhibited non-toxic activities against the Vero cells. Additionally, compound **1** further exhibited promising cytotoxic activity against both MCF-7 and HCT-116 cells, with IC_50_ values of 4.26 and 9.6 μM, respectively. Compound **1** significantly stimulated apoptotic breast cancer cell death resulting in a14.97-fold increase, with 40.57% of the cell population being arrested at the Pre-G1 cell cycle stage. Finally, its apoptosis-inducing activity was further validated through activation of BAX and caspase-9, and inhibition of BCL2 levels. Molecular docking experiments with the isolates showed a high affinity to Ras/MAPK when compared to rutin and sorafenib, and support the putative molecular mechanism of action of these compounds.

## Figures and Tables

**Table 1:** ^1^H-NMR (300 MHz) and ^13^C-NMR (100 MHz) in CD_3_OD spectra data of compound **1**.

Position	δ_H_ (ppm, *m*, *J* Hz)	δ_C_ (ppm)	Position	δ_H_ (ppm, *m*, *J* Hz)	δ_C_ (ppm)
2	5.47 (1H, *d*, *J* = 4.5 Hz)	81.8	9``		163.7
3	3.15 (1H, *m*)	49.8	10``		105.5
4		198.9	1```		129.1
5		165.4	2```, 6```	6.92 (2H, *d*, *J* = 8.4 Hz)	130.7
6	5.76 (1H, *d*, *J* = 2.1 Hz)	97.7	3```, 5```	6.79 (2H, *d*, *J* = 8.4 Hz)	116.9
7		168.6	4```		158.8
8	5.79 (1H, *d*, *J* = 2.1 Hz)	96.5	2````	4.96 (1H, *m*)	82.8
9		164.7	3````	5.89 (1H, *d*, *J* = 2.1 Hz)	97.8
10		104.2	4````		197.3
1`		129.5	5````		159.2
2`, 6`	7.11 (2H, *d*, *J* = 8.7 Hz)	128.9	6````	5.75 (1H, *s*)	96.6
3`, 5`	6.64 (2H, *d*, *J* = 8.4 Hz)	116.6	7````		168.5
4`		159.9	8````	5.75 (1H, *s*)	96.6
2``	5.14 (1H, *d*, *J* = 9.0 Hz)	83.5	9````		164.7
3``	3.32 (1H, *m*)	51.2	10````		103.3
4``		196.6	1`````		129.1
5``		165.7	2`````, 6`````	7.01 (2H, *d*, *J* = 8.4 Hz)	131.3
6``	5.87 (1H, *d*, *J* = 2.1 Hz)	97.5	3`````, 5`````	6.78 (2H, *d*, *J* = 8.7 Hz)	116.8
7``		168.6	4`````		159.9
8``	5.98 (1H, *m*)	96.9			

**Table 2:** In vitro cytotoxic effects (IC_50_, µM) of compounds **1**–**6**, **8** and **9** on HepG2 and Vero cell lines.

	Compound								
Cell line	1	2	3	4	5	6	8	9	Doxo
HepG2	8.6	12.3	29.8	27.2	9.4	32.5	15.1	32.6	7.7
Vero	56.6	>136.5	>141.9	123.5	111.6	>147.8	50.5	202.7	45.8

DOXO = Doxorubicin (positive control).

**Table 3:** The docking score and binding energy of the isolates and reference compound.

Compound	Docking Score (KJ mol^−1^)	Glide Emodel (Kcal mol^−1^)
**2**	−7.82	−59.02
**3**	−6.24	−51.75
**4**	−7.37	−59.42
**5**	−6.82	−58.55
**6**	−7.14	−58.90
**7**	−8.55	−58.78
**8**	−8.34	−100.70
**9**	−5.49	−31.27
**10**	−5.29	−45.43
**Rutin**	−7.32	−69.18
**Sorafenib**	−8.376	−81.119

## Data Availability

No new data were created or analyzed in this study. Data sharing is not applicable to this article. The data presented in this study are available in Supplementary Materials.

## Supplementary Materials

The following are available online, Figures S1–S7: Mass and NMR Spectra of Compound **1**, Figures S8–S13: 2D of ligand receptor interaction of MAPK ligand, compound **6**, compound **7**, compound **8**, rutin, and Sorafenib in MAPK active domain (PDB 5EKN) shown hydrogen bonding, hydrophilic and electrostatic interaction.

## Sample Availability

Samples of the compounds are available from the authors.
