# Supplementary material for: New Antiproliferative Triflavanone from Thymelaea hirsuta—Isolation, Structure Elucidation and Molecular Docking Studies
Source: Molecules. 2021 Jan 31;26(3):739. doi: 10.3390/molecules26030739 (PMC7867015; doi:10.3390/molecules26030739)
Supplement: Supplementary file 1 [file molecules-26-00739-s001.pdf]

New Antiproliferative Triflavanone from *Thymelaea hirsuta* – Isolation, Structure Elucidation and Molecular Docking Studies

Sameh S. Elhady [1,†], Reda F. A. Abdelhameed [2,†], Mayada M. El-Ayouty [2,3], Amany K. Ibrahim [2], Eman S. Habib [2], Mohamed S. Elgawish [4], Hashim A. Hassanean [2], Martin K. Safo [5], Mohamed S. Nafie [6] and Safwat A. Ahmed [2,*]

[1] Department of Natural Products, Faculty of Pharmacy, King Abdulaziz University, Jeddah 21589, Saudi Arabia; ssahmed@kau.edu.sa (S.S.E.)
[2] Department of Pharmacognosy, Faculty of Pharmacy, Suez Canal University, 41522 Ismailia, Egypt; omarreda_70@yahoo.com (R.F.A.A.); maya.badawy159@gmail.com (M.M.E.); am_kamal66@yahoo.com (A.K.I.); emy_197@hotmail.com (E.S.H.); hashem_omar@pharm.suez.edu.eg (H.A.H.); safwat_aa@yahoo.com (S.A.A.)
[3] Department of Pharmacognosy, Faculty of Pharmacy, Sinai University, El-Arish 45511, Egypt
[4] Department of Medicinal Chemistry, Faculty of Pharmacy, Suez Canal University, 41522 Ismailia, Egypt; mohamed_elgawish@pharm.suez.edu.eg (M.S.E.)
[5] Department of Medicinal Chemistry, School of Pharmacy and Institute for Structural Biology, Drug Discovery and Development, Virginia Commonwealth University, Richmond, VA 23219, USA msafo@vcu.edu (M.K.S.)
[6] Department of Chemistry, Faculty of Science, Suez Canal University, Ismailia 41522, Egypt; mohamed_nafie@science.suez.edu.eg (M.S.N.)
[†] These authors equally contributed to this work
[*] Correspondence: safwat_aa@yahoo.com (S.A.A.); Tel.: (+20) 010-92638387, Fax: (+20) 064-3230741

Additional Experimental Detail

1) **Figure S1.** LC-MS/MS of compound **1**
2) **Figure S2.** ^{1}H-NMR spectrum of compound **1** (CD_3OD, 300 MHz)
3) **Figure S3.** Partial expansions of the ^{1}H-NMR spectrum of compound **1** (CD_3OD, 300 MHz)
4) **Figure S4.** Partial expansions of the ^{1}H-NMR spectrum of compound **1** (CD_3OD, 300 MHz)
5) **Figure S5.** ^{13}C-NMR spectrum of compound **1** (CD_3OD, 100 MHz)
6) **Figure S6.** 2D-HMBC spectrum of compound **1** (CD_3OD, 300 MHz)
7) **Figure S7.** 2D-HMBC spectrum of compound **1** (CD_3OD, 300 MHz)
8) **Figure S8.** 2D of ligand receptor interaction of MAPK ligand in MAPK active domain (PDB 5EKN) shown hydrogen bonding, hydrophilic and electrostatic interaction
9) **Figure S9.** 2D of ligand receptor interaction of compound **6** in MAPK active domain (PDB 5EKN) shown hydrogen bonding, hydrophilic and electrostatic interaction
10) **Figure S10.** 2D of ligand receptor interaction of compound **7** in MAPK active domain (PDB 5EKN) shown hydrogen bonding, hydrophilic and electrostatic interaction
11) **Figure S11.** 2D of ligand receptor interaction of compound **8** in MAPK active domain (PDB 5EKN) shown hydrogen bonding, hydrophilic and electrostatic interaction
12) **Figure S12.** 2D of ligand receptor interaction of Rutin in MAPK active domain (PDB 5EKN) shown hydrogen bonding, hydrophilic and electrostatic interaction
13) **Figure S13.** 2D of ligand receptor interaction of Sorafenib in MAPK active domain (PDB 5EKN) shown hydrogen bonding, hydrophilic and electrostatic interaction

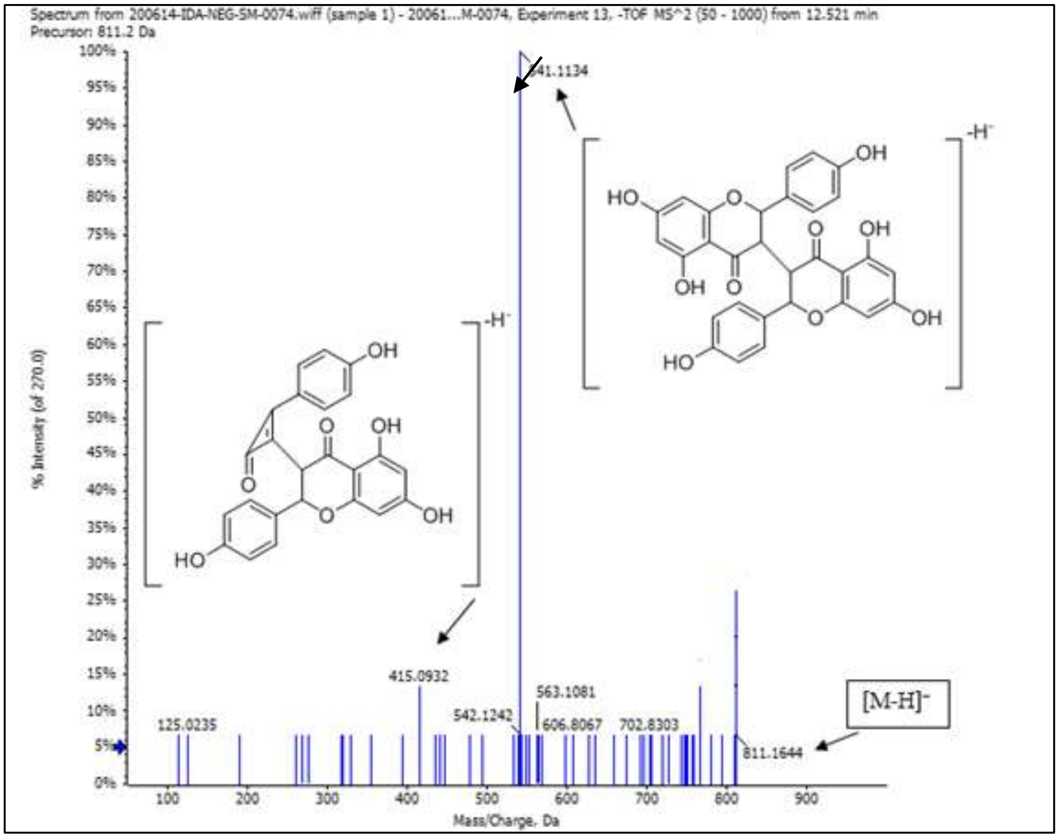

Figure S1: LC-MS/MS of compound **1**

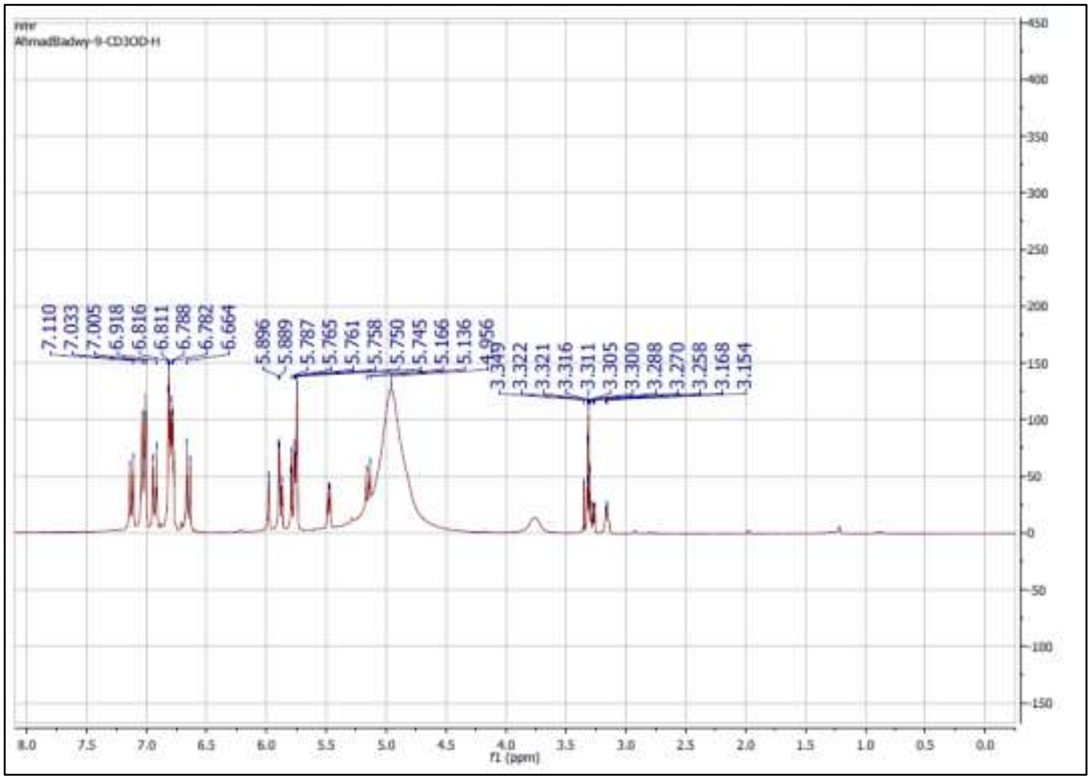

Figure S2: ^{1}H-NMR spectrum of compound **1** (CD_3OD, 300 MHz)

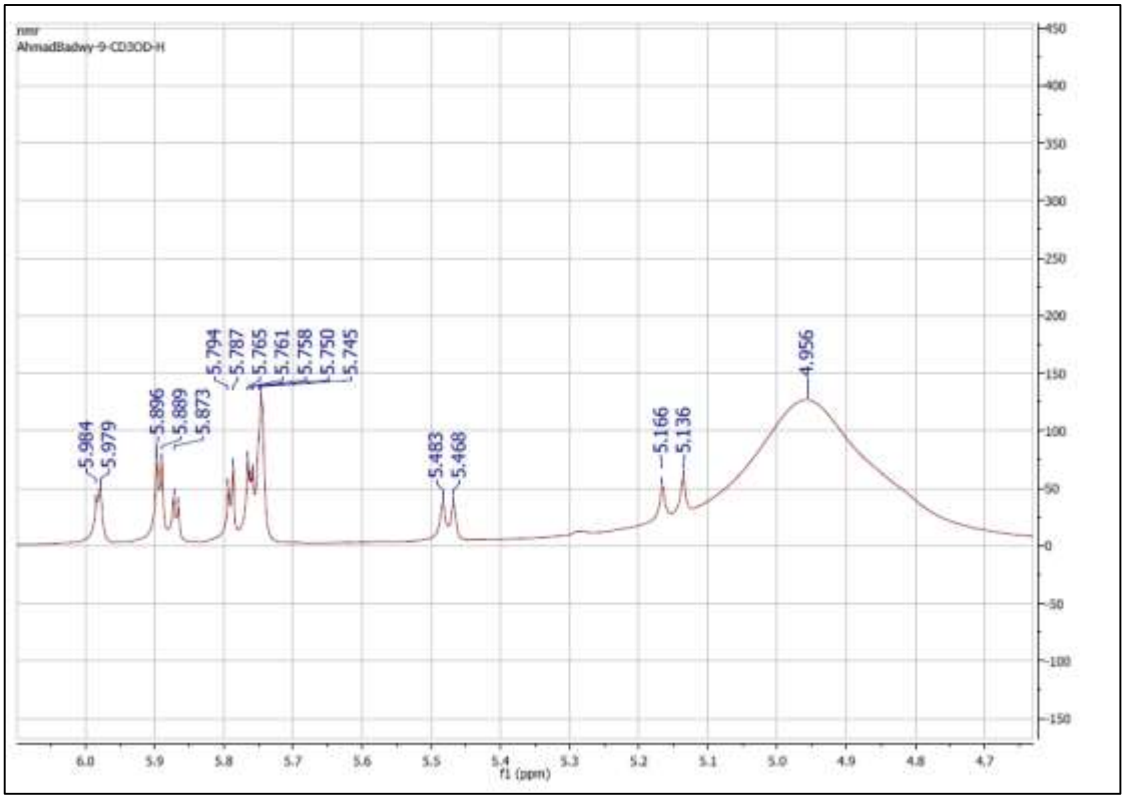

Figure S3: Partial expansions of the ^{1}H-NMR spectrum of compound **1** (CD_3OD, 300 MHz)

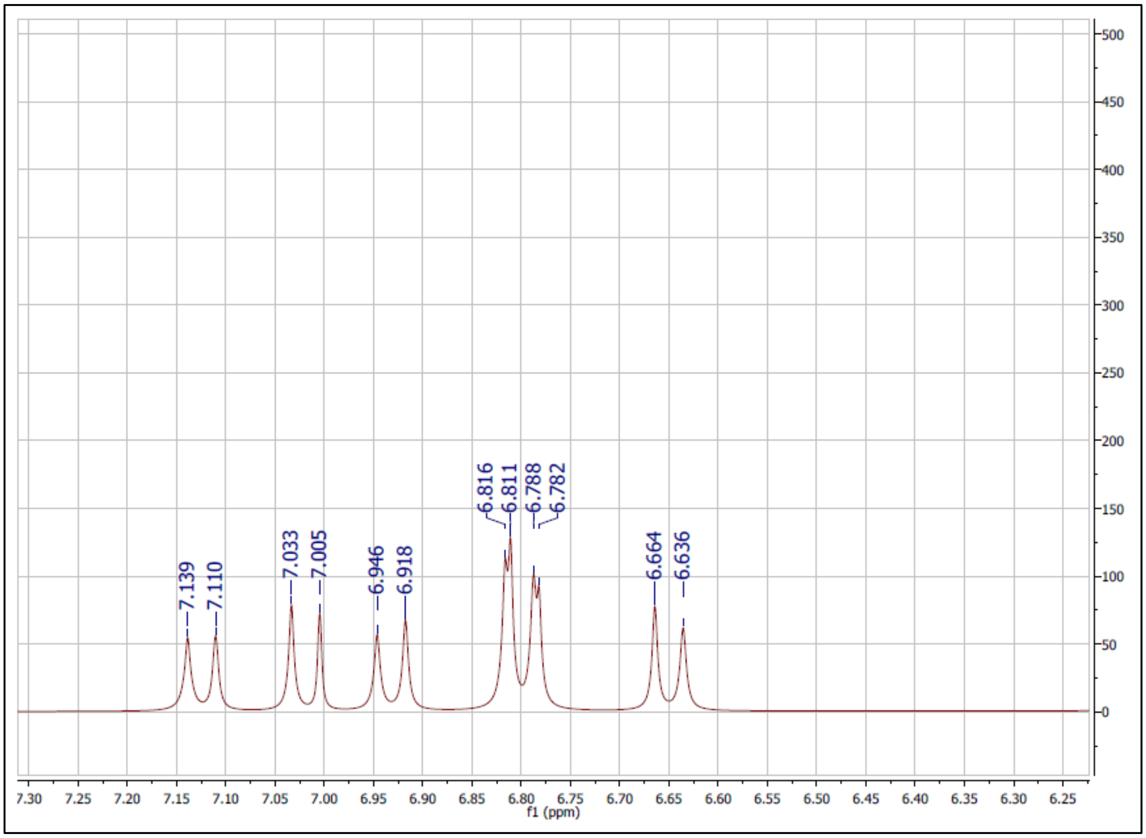

Figure S4: Partial expansions of the ^{1}H-NMR spectrum of compound **1** in (CD_3OD, 300 MHz)

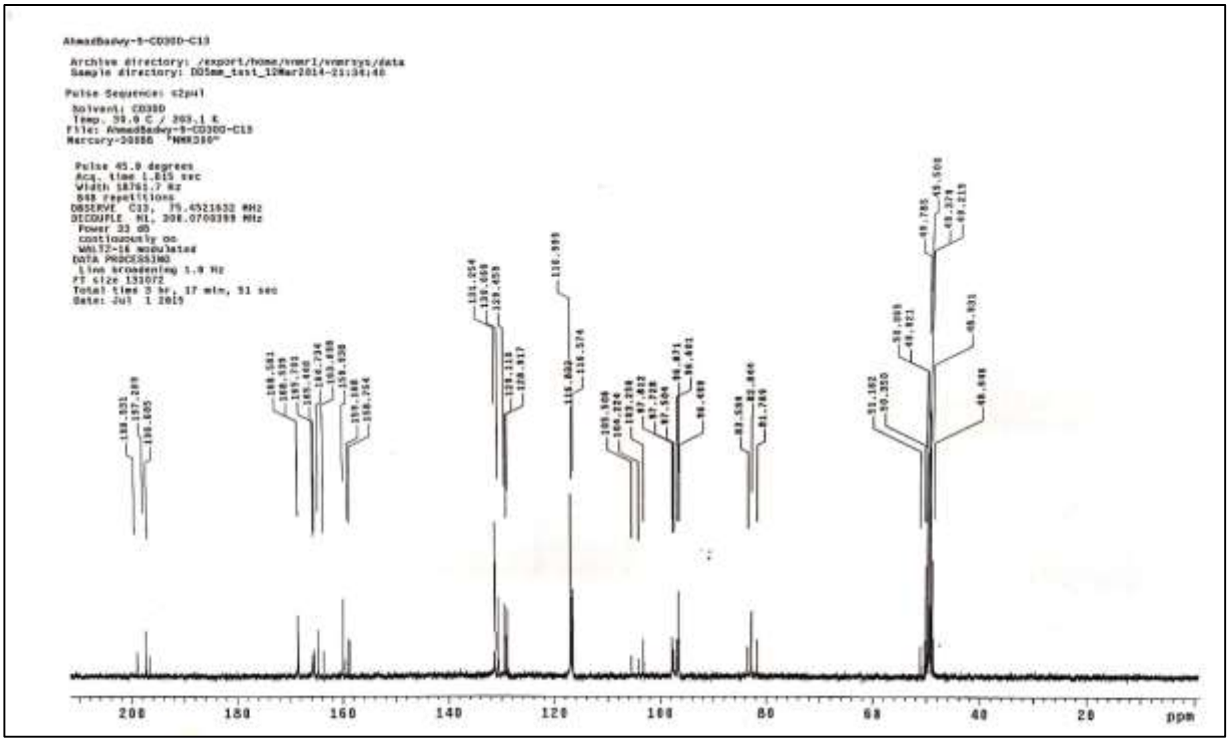

Figure S5: ^{13}C-NMR spectrum of compound **1** in (CD_3OD, 100 MHz)

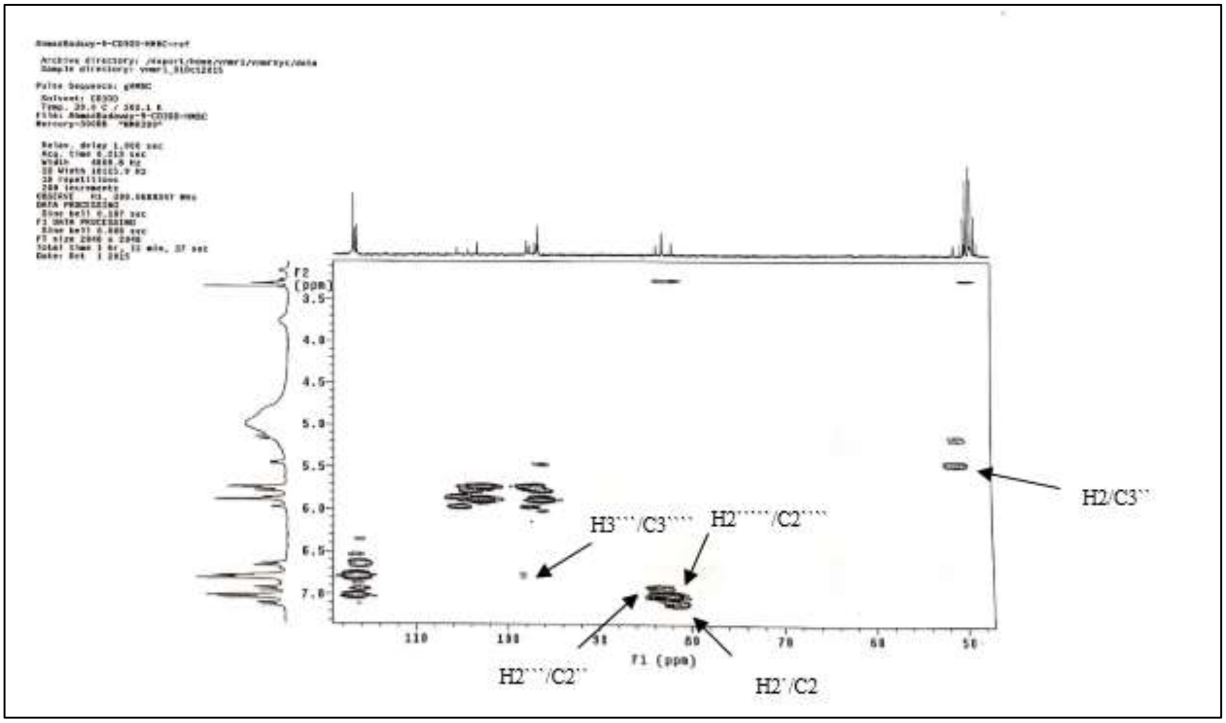

Figure S6: 2D-HMBC spectrum of compound **1** in (CD_3OD, 300 MHz)

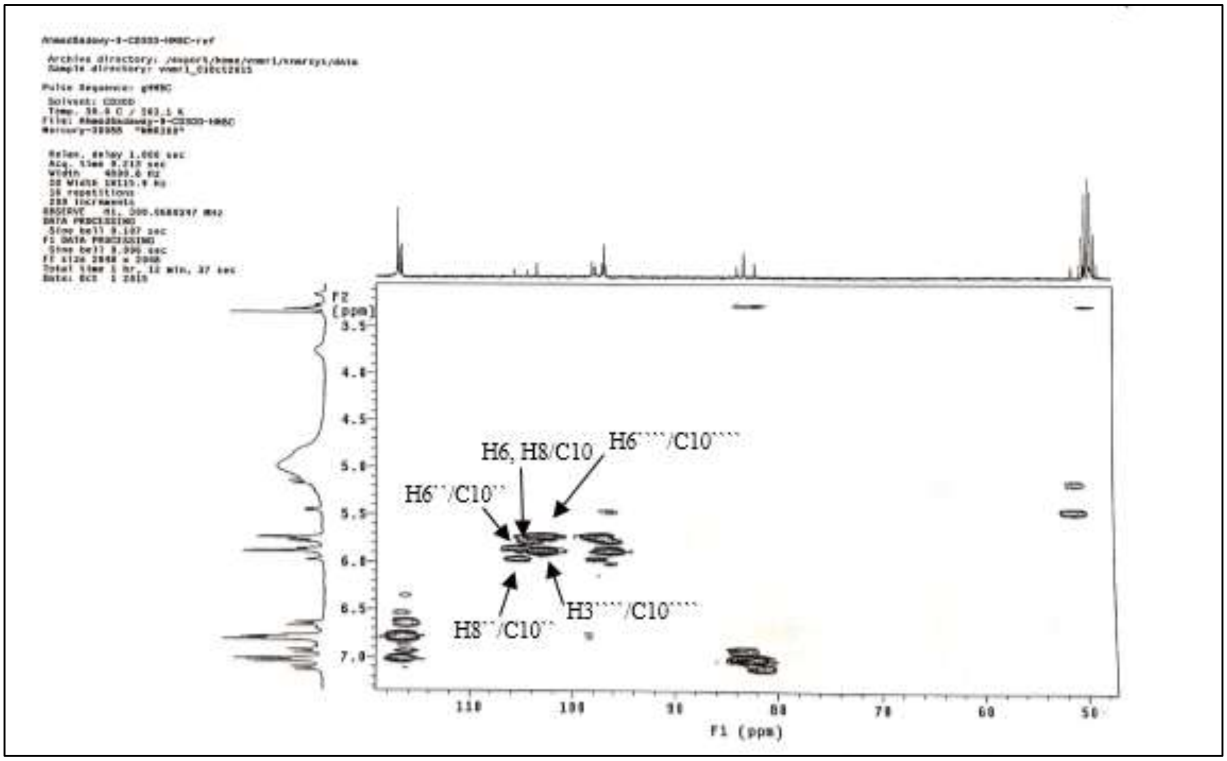

Figure S7: 2D-HMBC spectrum of compound **1** in (CD_3OD, 300 MHz)

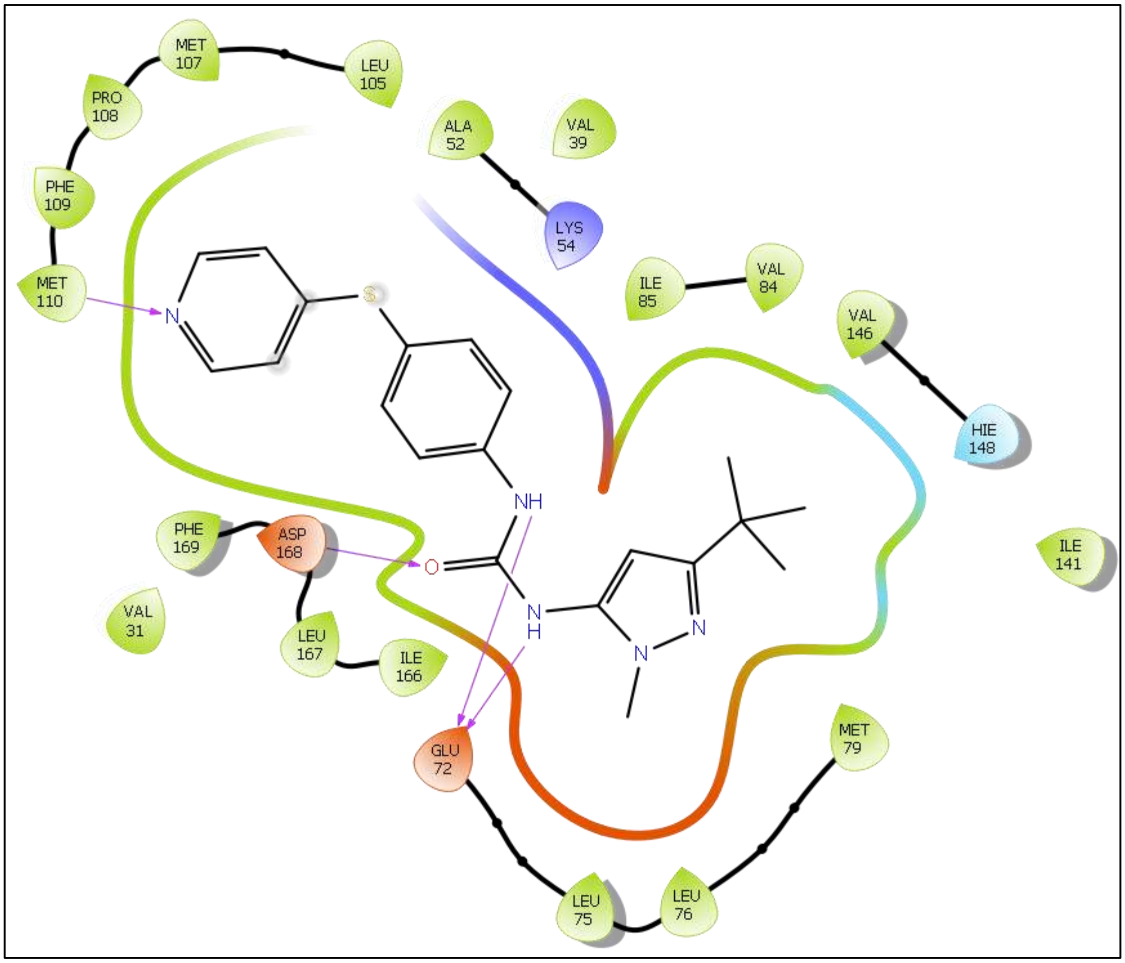

Figure S8. 2D of ligand receptor interaction of MAPK ligand in MAPK active domain (PDB 5EKN) shown hydrogen bonding, hydrophilic and electrostatic interaction

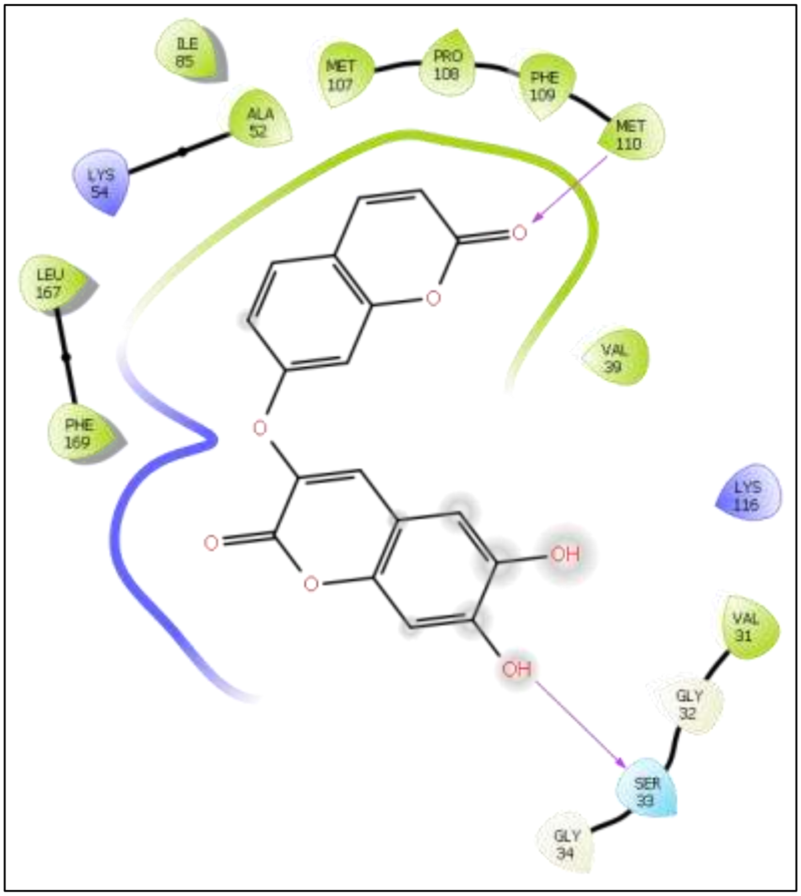

Figure S9. 2D of ligand receptor interaction of compound **6** in MAPK active domain (PDB 5EKN) shown hydrogen bonding, hydrophilic and electrostatic interaction

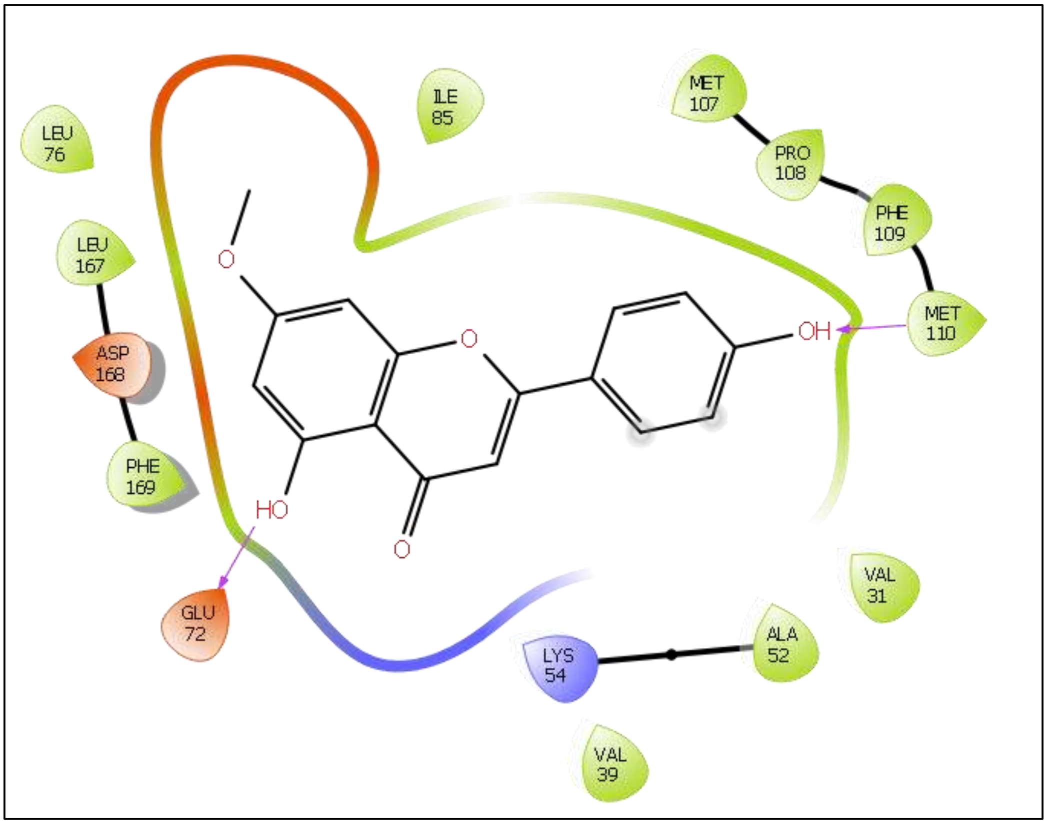

Figure S10. 2D of ligand receptor interaction of compound **7** in MAPK active domain (PDB 5EKN) shown hydrogen bonding, hydrophilic and electrostatic interaction

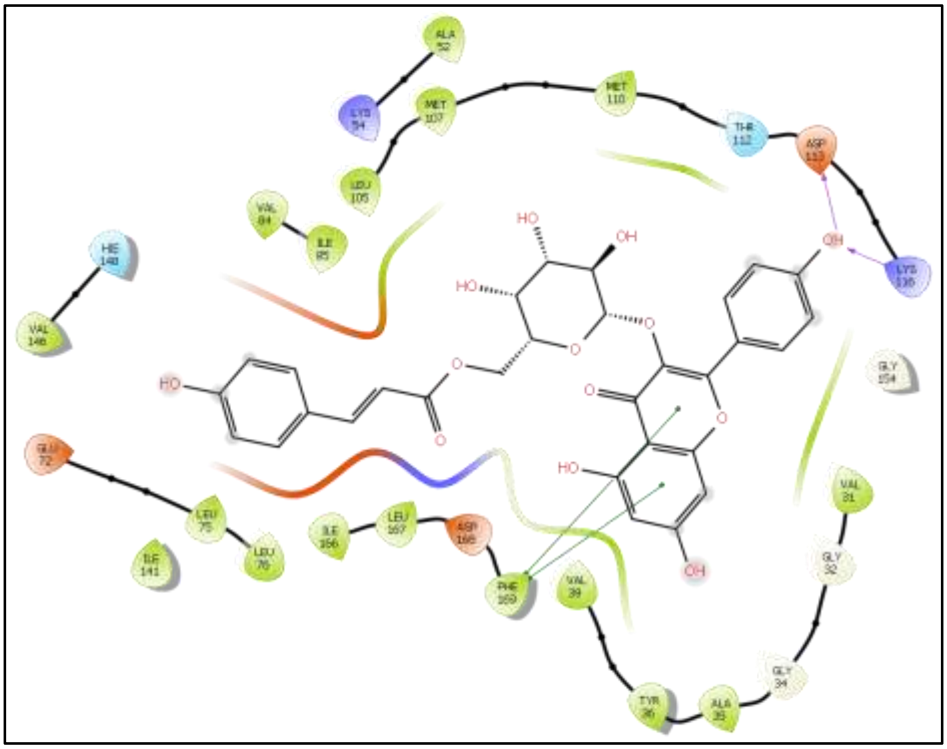

Figure S11. 2D of ligand receptor interaction of compound **8** in MAPK active domain (PDB 5EKN) shown hydrogen bonding, hydrophilic and electrostatic interaction

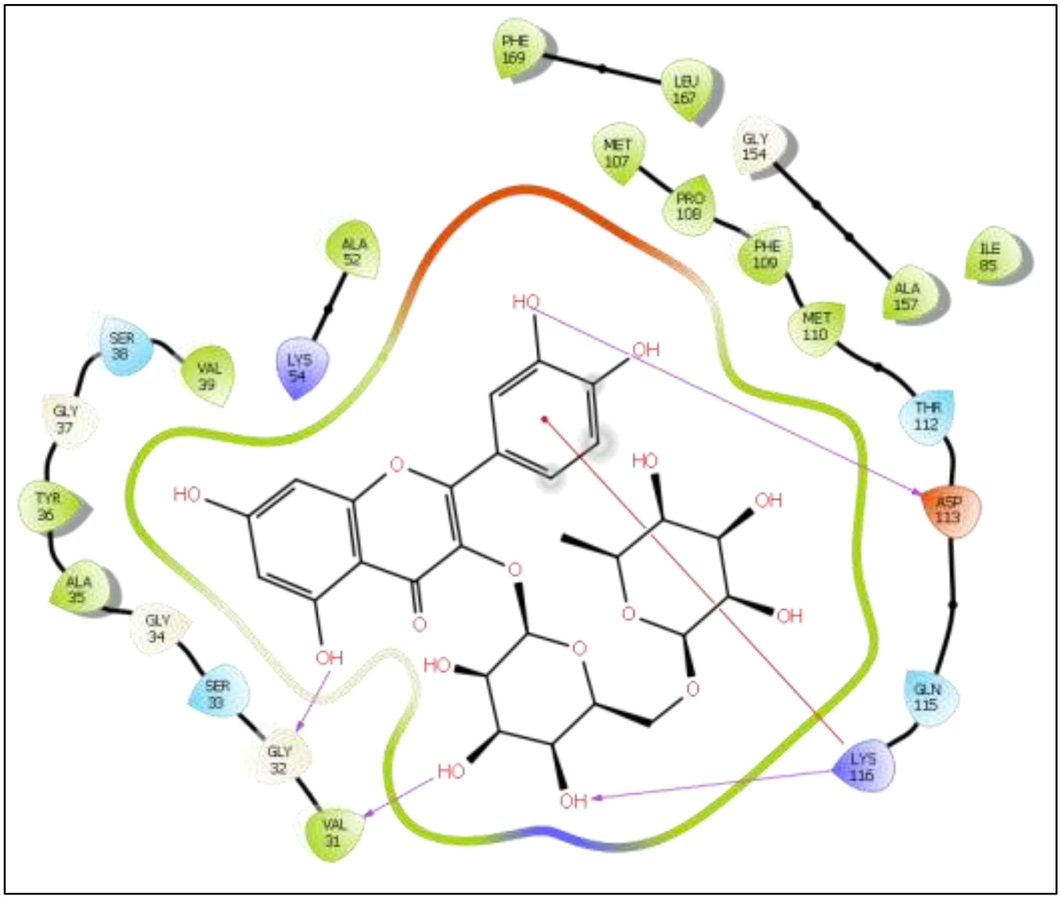

Figure S12. 2D of ligand receptor interaction of Rutin in MAPK active domain (PDB 5EKN) shown hydrogen bonding, hydrophilic and electrostatic interaction

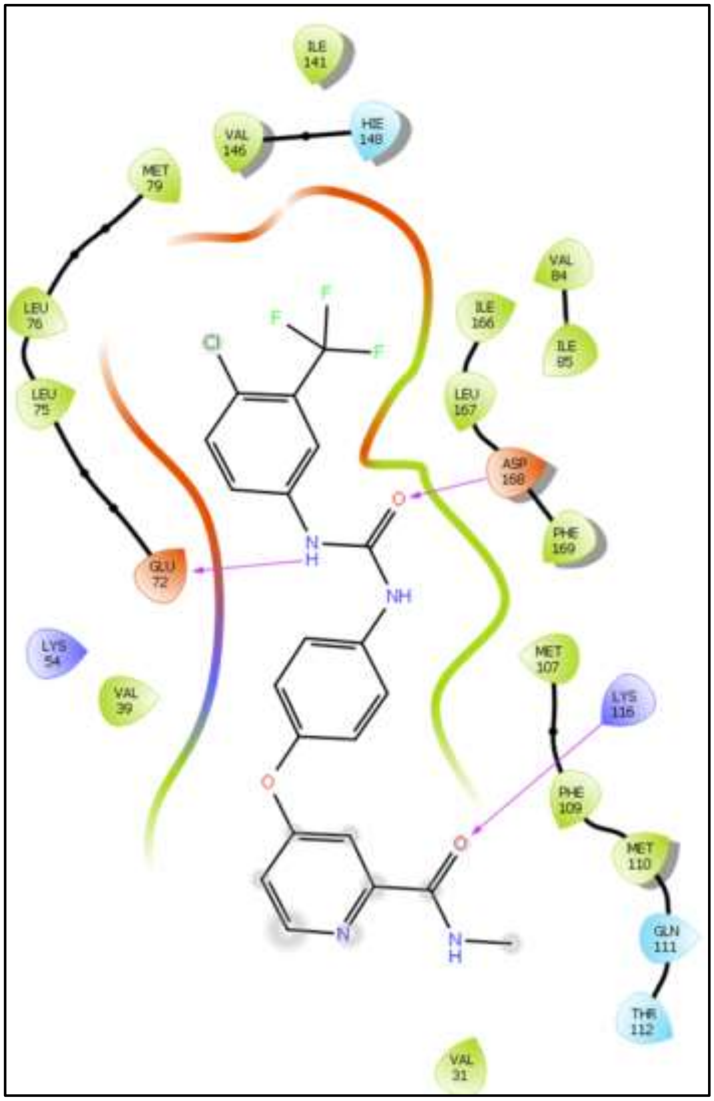

Figure S13. 2D of ligand receptor interaction of Sorafenib in MAPK active domain (PDB 5EKN) shown hydrogen bonding, hydrophilic and electrostatic interaction
